# Haplotype analysis of *BRCA1* intragenic markers in Iranian patients with familial breast and ovarian cancer

**Published:** 2016-04

**Authors:** Seyed Mohsen Miresmaeili, Dor Mohammad Kordi Tamandani, Seyed Mehdi Kalantar, Seyed Mohammad Moshtaghioun

**Affiliations:** 1 *Department of Biology, Faculty of Science, University of Sistan and Baluchestan, Zahedan, Iran.*; 2 *Research and Clinical Center for Infertility, Shahid Sadoughi University of Medical Sciences, Yazd, Iran.*; 3 *Department of Biology, Faculty of Sciences, University of Yazd, Yazd, Iran.*

**Keywords:** *Breast cancer*, *Ovarian cancer*, *STR haplotyping*

## Abstract

**Background:**

Breast cancer is the most common malignancy in women. Breast Cancer Type 1 Susceptibility gene (*BRCA1*) is a tumor suppressor gene, involved in DNA damage repair and in 81% of the breast-ovarian cancer families were due to *BRCA1*. In some clinically investigated genes, the intragenic marker polymorphism is important and the screening of such mutations is faster by using short tandem repeat (STR) polymorphism. Individual polymorphism of STR is a good evidence for following inheritance of repeat polymorphism.

**Objective:**

The aim of this study was to evaluate three intragenic *BRCA1* marker polymorphisms in families, which have two or more patients with breast/ovarian cancer in comparison to healthy women.

**Materials and Methods:**

A total of 107 breast and/or ovarian cancer patients and 93 unrelated healthy women with no clinical phenotype of any malignancy or familial cancer history constitute the study groups. Haplotyping analysis, at 3 intragenic *BRCA1* microsatellite markers (D17S855, D17S1322 and D17S1323), were performed for all subject and control groups using labeled primers.

**Results:**

After fragment analysis, significance differences were observed as follows: two alleles of D17S855; allele 146 (p=0.02) and 150 (p=0.006), and two alleles of D17S1322, allele 121 (p=0.015) and 142 (p=0.043). These differences were compared with control group. There was significance difference in 8 di/tri allelic haplotypes in present experimental subjects. Some haplotypes were observed to have approximately twice the relation risk for breast cancer.

**Conclusion:**

According to recent results, assessment of presence or absence of mentioned alleles in *BRCA1* microsatellite can be used for prognosis in individuals, suspected of having or not having the breast cancer.

## Introduction

Breast cancer is responsible for a large amount of mortality rate in women worldwide. Hereditary Breast and Ovarian Cancer is a syndrome that increases the lifetime risk for developing breast and/or ovarian cancer and other malignancies in women. Inheritance of this syndrome is usually based on germ-line mutation in one allele of either the Breast Cancer Type 1 Susceptibility (*BRCA1*) or Breast Cancer Type 2 Susceptibility (*BRCA2*) genes (1-4). In American society, more than 1.5 million new breast cancer cases occurred in 2014 (5). In Iranian society, lifetime prevalence of breast cancer is one in eight female cases and the breast cancer patients are younger than those in advanced countries as well (6). About 15% of women with familial breast cancer are implicated to have mutation in *BRCA1* and *BRCA2* genes, also, all women with ovarian cancer are affected by some mutation in these genes (7).

The *BRCA1* was first identified by linkage analysis on chromosome 17q21 in 1994 (2). An autosomal hereditary pattern is linked to *BRCA1* in some cases (8). *BRCA1* gene consists of 24 exons that encode a protein with 1863 amino acids with three major functional domains and a ubiqitin ligase domain named as: RING domain; at the N-terminus, nuclear localization signal domain (NLS) in the middle and BRCT (BRCA1 C-terminal domain at C-terminus, respectively (9). Familial mutation may be hereditary, while screening and detection of mutations in *BRCA1* gene may help in medical management of patients and their families. *BRCA1* haplotype analysis was carried out to estimate the ancestral origin of mutations and haplotypes associated with particular diseases (10, 11). Founder mutation analysis performed using haplotype assessment to find the first carrier of mutation, in several population founder mutation have been originated (12-16). Genetic polymorphism of tumor suppressor and DNA repair genes have been involved in breast cancer risk (17).

In some clinically investigated genes, the intragenic marker polymorphism is the most important. In screening of large genes, it is difficult to select patients carrying such mutations (18). Screening of *BRCA1* mutation is carried out much faster by using short tandem repeat (STR) polymorphism. Individual polymorphism of STR is a good evidence of following the inheritance of repeat polymorphism. Several markers exist within the flanking *BRCA1*. D17S1323 (intron 12), D17S1322 (intron 19), and D17S855 (intron 20); were genotyped at the 3′ end of *BRCA1* gene. D17S855 and D17S1323 are di-nucleotide STR and D17S1322 is a tri-nucleotide repeat (19, 20). 

Our main goal was to evaluate haplotyping of three intragenic markers of *BRCA1* between breast cancer patients and healthy women.

## Materials and methods

This case-control study was carried out in Science and Arts University, Yazd, Iran. The study protocol was approved by the Ethics Committee of Shahid Sadoughi University of Medical Sciences, Yazd, Iran. Written informed consent was obtained from all participants.

107 women aged 36-51 years with breast or ovarian cancer who were admitted to Yazd hospitals between January 2014 to July 2015 were enrolled as case group. Diagnosis was confirmed in all patients with pathologic findings. Information regarding family history of cancer and other relevant clinical details was available in case group. 

The control-group members (n=93) were age-matched unrelated normal healthy women that inhabit in yazd and had no history of breast cancer or other cancers in their family members. Peripheral blood samples were collected in Ethylenediaminetetraacetic acid (EDTA) from all participants. Genomic DNA was extracted from peripheral blood using Prime Prep kit (Cat.k-2000, Genet Bio, Korea) according to the manufacturer′s protocol.

**PCR program**

The genomic intragenic amplicons of di/tri- nucleotides of *BRCA1* genes were individually amplified by PCR procedure. For PCR amplification, three sets of primer pairs were used to amplify three intragenic markers of *BRCA1* gene. PCR amplification for a 25 µL PCR reaction volume was used. The PCR mixture consisted of 12.5µL of Taq 2X Master Mix RED (cat.A180301, Ampliqon), 9.5 µL of water and 2.0 µL of primer mix. To make the final reaction volume, 1.0 µL DNA was also added. The PCR reaction was run with an initial denaturation at 94^o^C for 4 min, followed by 32 cycles of denaturation at 94^o^C for 30 sec, while the annealing temperature was specific for each primer for 30 sec and the extension at 72^o^C for 1 min. Final extension was carried out for 10 min at 72^o^C. Primers were designed by Sci-Ed Software (Table I) (21). 

**Fragment analysis**

For STR analysis, forward primers of each pair of primers were labeled with FAM, HEX and TAMRA for D17S1322, D17S1323 and D17S855, respectively. The samples were read on ABI Prism 3730xl using Genemarker Software (version 2.6.3).

**Statistical analysis**

All statistical analyses were carried out using the IBM SPSS (version 22.0) software. A non-parametric ^2^ test was used to compare the differences between two groups. Statistical significance was accepted at p<0.05.

## Results

Haplotype assessments were carried out by fragment analysis. Haplotype analysis was performed with 3 intragenic polymorphic markers (D17S855, D17S1322 and D17S1323), located in introns 20, 19 and 12, respectively. STR heliotyping was carried out using fragment analysis. It was observed that D17S1323 had 11 allels (Table II) and D17S855 had 11 alleles (Table III). Also, D17S1322 had 13 alleles (Table VI). There was significant difference in two alleles of D17S855: allele 146 (p=0.02) and 150 (p=0.006); and two allele of D17S1322: allele 121 (p=0.015) and 142 (p=0.043), as compared to control group.

**Table I:** Primer sequences for *BRCA1* haplotype analysis

**Marker**	**Forward primer**	**Reverse primer**	**Annealing (** ^o^ **C)**	**Product size (bp)**
D17S1322	5′CTAGCCTGGGCAACAAACGA	5′GCAAGCAGGAATGGAAC	58	134
D17S1323	5′TAGGAGATGGATTATTGGT	5′AAGCAACTTTGCAATGAGT	55	150
D17S855	5′GGATGGCCTTTTAGAAAGTGG	5′ACACAGACTTGTCCTACTGCC	58	151

**Table II:** Allele distribution of the D17S1323 in Iranian women with breast/ovarian cancer

	**No. of samples**	**No. of alleles**	**Allele size, base pairs (bp)**										
			140	144	146	148	150	152	154	156	158	162	164
Breast cancer	107	214	1	1	2	10	97	12	10	55	21	3	2
% of alleles			0.5	0.5	0.9	4.7	45.3	5.6	4.7	25.7	9.8	1.4	0.9
p-value			0.54	0.54	0.1	0.56	0.17	0.44	0.29	0.54	0.55	0.17	0.27
Healthy women	93	186	0	0	6	9	75	12	12	48	18	6	0
% of alleles			0	0	3.2	4.8	40.3	6.5	6.5	25.8	9.7	3.2	0

**Table III:** Allele distribution of the D17S855 in Iranian women with breast/ovarian cancer (p-value with significance is in bold

	**No. of samples**	**No. of alleles**	**Allele size, base pairs (bp)**										
			136	138	140	142	144	146	148	150	152	154	156
Breast cancer	107	214	4	2	2	21	69	37	40	28	7	0	4
% of alleles			0.08	0.9	0.9	9.8	32.2	17.3	18.7	13.1	0.33	0	1.9
p-value			1.9	0.27	0.43	0.35	0.17	0.02	0.46	0.006	0.36	0.1	0.3
Healthy women	93	186	0	0	3	21	51	18	33	43	8	3	6
% of alleles			0	0	1.6	11.3	27.4	9.7	17.8	23.1	4.3	1.6	3.2

**Table VI:** Comparison of other prevalence haplotypes in breast cancer patients and control group

**Haplotype (D17s855, D17s1323, D17s1322)**	**Patients (N=107)**	**Control (N=93)**
144, 150, 112	34 (31.7%, p=0.03, RR=1.33)	18 (19.4%)
144, 152,112	5 (4.7%, p=0.04, RR=1.91)	0
144, 156, 112	22 (20.5%, p=0.01, RR=1.5)	8 (8.6%)
148, 150, 121	9 (8.4%, p<0.01, RR=1.99)	0
146, 150, 112	29 (27.1%, p=0.017, RR=1.4)	13 (14%)
146, 150, 118	16 (14.9%, p=0.04, RR=1.42)	6 (6.5%)

a Prevalence of haplotype in percentage

b Chi-square, with statistical significance if p<0.05

c Relative risk

## Discussion

In this study, the haplotyping of intragenic markers of *BRCA1* were evaluated. Main conclusion of this study is that four haplotypes can be evaluated between breast cancer patients and healthy women for D17S855 and D17S1322 respectively, 1) 146, 121; 2) 146, 142; 3) 150, 121; 4) 150, 142. Two out of four possible haplotypes were significant in patients, which showed increased risks for breast/ovarian cases with either of these two haplotypes (RR of 1.6 and 1.92 for both haplotypes, respectively) (p<0.01). The proportion of 121 and 150 haplotypes in experimental subjects were approximately twice in numbers when compared with the prevalence in controls. Also, other tri-allelic haplotypes in patients had significant relative risk. As illustrated in table IV, the proportion of 144, 152, 112 and 148, 150, 121 haplotypes in studied patients were approximately twice in number when compared with prevalence in controls. 

Nowacka-Zawisza *et al* showed that polymorphism of di-nucleotide CA repeat at *RAD51* and *BRCA2* gene regions might be associated with genetic susceptibility to breast cancer (20). Also, in another study, *BRCA1* mutations were defined by presence of *BRCA1* intragenic STR markers. de la Hoya *et al* suggested that mutation on *BRCA1* were correlated with two alleles of D17S855 (139 and 141bp) in Spanish population (22).

While, our study indicated that in Iranian population, the breast cancer susceptibility is not randomly distributed but it is clustered in subset of *BRCA1* alleles that can be identified by D17S855 and D17S1322 genotyping. Osorio *et al *observed one specific allele for microsatellite marker D17S855, which are also more frequently associated with *BRCA1* mutations (18). In Malaya patients*BRCA1* was found to have a deleterious mutation, c.2845insA, which was associated with STR haplotype, 142-127-159 for D17S855, D17S1322 and D17S323, respectively (11). 

The *BRCA1* mutation at c.5266dupC is originally a founder mutation in the Ashkenazi Jewish population, and same mutation is also found in several different population groups such as Russian, Latvian, Ukrainian, Czech, Slovak, Polish, Danish, Dutch, French, German, Italian, Greek, Brazilian and Azerbaijan. Haplotyping by microsatellite markers confirmed that all mutation carriers shared a common *BRCA1* STR haplotype (10, 23).

## Conclusion

In conclusion, screening of microsatellite polymorphism of DNA repairing genes in families with breast cancer history can be useful tool for prognosis, incidence and occurrence of breast cancer in healthy women/men. Therefore, assessment of presence or absence of mentioned haplotype in the *BRCA1* microsatellites can be used for prognosis in individuals, suspected of having or not having breast cancer. 

## Note

***This article extracted from Ph.D. Thesis. (Seyed Mohsen Miresmaili)***
